# ‘Endless variation on a theme’: a document analysis of international and UK major trauma triage tools

**DOI:** 10.29045/14784726.2024.12.9.3.28

**Published:** 2024-12-01

**Authors:** Gordon Fuller, Chris Holt, Samuel Keating, Janette Turner, MATTS research group contributors

**Affiliations:** University of Sheffield ORCID iD: https://orcid.org/0000-0001-8532-3500; University of Sheffield; University of Sheffield; University of Sheffield ORCID iD: https://orcid.org/0000-0003-3884-7875; (see Acknowledgements)

**Keywords:** document analysis, injuries, major trauma, triage, triage tools

## Abstract

**Introduction:**

Triage tools are used within trauma networks to identify which injured patients should be bypassed and pre-alerted to major trauma centres. Despite the importance of treating the ‘right patient in the right place at the right time’, there has been no consensus on triage tool structure or content. This study aimed to identify, collate, review, summarise and recognise patterns across established major trauma triage tools.

**Methods:**

UK and international triage tools used between 2012 and 2021 were identified through literature review and correspondence with trauma networks. A conceptual content analysis was then undertaken using an inductive codebook, comprising concepts of triage tool structure, intended population, inclusion criteria and included variables and thresholds. Thematic analysis was also performed to identify higher-level patterns within the data, with emerging patterns becoming categories for analysis. A narrative synthesis of findings was then undertaken.

**Results:**

In total, 53 major trauma tools were identified, comprising 19 UK tools and 35 published international tools. Most triage tools (n = 42/53, 80%) were developed by expert opinion, were paper based and shared a common structure of multiple domains, with constituent triage predictors assessed in parallel. A minority of tools were statistically derived prediction models, operationalised either as simple scores (n = 10, 19%) or as an electronic application (n = 1, 1%). Overall, 173 distinct triage variables were used, with the median number of constituent triage variables per triage tool being 19 (range 3–31). Four distinct patterns of triage tools were identified during thematic analysis, which differed in terms of format, number of triage variables, thresholds, scope for clinical judgement and relative diagnostic accuracy.

**Conclusion:**

Many diverse major trauma triage tools were identified, with no consensus in format, structure or content. Quantification of constituent variables and identification of distinct categories of triage tools may guide the design of future triage tools.

## Introduction

There are an estimated 20,000 cases of major trauma each year in England, resulting in 5400 deaths and 8000 severe disabilities (Kehoe et al., 2015; National Audit Office, 2017). Historically major trauma predominated in younger adults, resulting from high-energy mechanisms of injury or penetrating trauma. More recently incidence has increased in older age groups, with a typical major trauma case now occurring following a ground-level fall in an elderly patient (Coats & Lecky, 2017). Although major trauma is a prominent public health issue, it has low prevalence. In the year spanning 2018‒2019, there were 11.7 million contacts via 999 with the ambulance service, of which approximately 3.5% represented injuries, but only an estimated 0.002% received major trauma working impression codes (Henderson et al., 2019; Noble et al., 2022).

In 2012, care of injured patients in England was reconfigured with the introduction of regional major trauma networks, aiming to match injury severity with hospital capability (McCullough et al., 2014). These consist of a centralised major trauma centre (MTC) serving a defined catchment area, providing all the clinical specialties required for the acute management and rehabilitation of the most seriously injured patients (Vondy & Willett, 2011). MTCs correspond to American College of Surgeons (ACS) designated Level I or II trauma centres. Regional trauma networks also include trauma units (TUs), equivalent to ACS Level III or IV trauma centres, which are resourced to care for less severely injured cases, and local emergency hospitals (LEHs), which do not routinely receive acute trauma patients (McCullough et al., 2014). MTC care has been associated with decreased patient mortality in severely injured patients (Moran et al., 2018).

Trauma systems follow one of two broad conceptual models, referred to as ‘inclusive’ and ‘exclusive’ (McCullough et al., 2014). In exclusive systems, all patients with possible major trauma within the area covered by the MTC will be primarily transferred there from the scene, bypassing all other facilities. Conversely, the principle in inclusive systems is that TUs must be able to receive (and subsequently transfer if necessary) appropriate major trauma patients not initially requiring critical trauma interventions. The National Institute for Health and Care Excellence (NICE) major trauma service delivery guidelines recommend implementation of inclusive trauma networks in England (NICE, 2016).

Pre-hospital ambulance service triage tools are the key intervention within regional trauma networks to match patient need with hospital provision. They will identify which patients injured within the catchment areas of TUs and LEHs should be bypassed to MTCs (NICE, 2016). Furthermore, relevant to patients injured in both MTC and non-MTC catchment areas, triage tools are also used to inform MTC emergency department pre-alert calls, facilitating patient reception into resuscitation areas and activation of multidisciplinary hospital trauma teams (NICE, 2016).

Systematic reviews suggest that a wide range of triage tools are currently used with sub-optimal accuracy (Pandor et al., 2022; van Rein et al., 2017; van Rein et al., 2018; Voskens et al., 2017). However, despite the importance of treating the right person in the right place at the right time, triage tool structure and content has not been systematically examined. The aim of this study was to identify, collate, review and interpret established major trauma triage tools. Specific objectives were to identify all UK and published international triage tools, summarise structure and content and recognise patterns across triage tools.

## Methods

A qualitative document analysis was undertaken according to methodological best practice recommendations (Dalglish et al., 2020). First, major trauma triage tools were identified by a comprehensive strategy encompassing MEDLINE searches for systematic reviews of major trauma triage tools (Supplementary 1); direct email correspondence with UK trauma networks, ambulance services, the English National Clinical Director for Major Trauma and the Joint Royal Colleges Ambulance Liaison Committee (JRCALC); and ad hoc web searches for publicly available triage tools. This strategy aimed to identify all UK triage tools used between 2012 and 2021, corresponding to the time when trauma networks were implemented in the NHS, and published international major trauma triage tools from the same period. Pre-hospital scales and scores not implemented clinically or used primarily for research or prognosis were not eligible. Non-major trauma pre-hospital triage tools, tools specific to individual injuries or tools primarily for burns were also excluded from consideration.

Document analysis then proceeded in three stages, with skimming (superficial examination), reading (thorough examination) and interpretation of identified tools (Dalglish et al., 2020). A conceptual content analysis was undertaken using an inductive codebook developed by the research team after initial skimming of identified triage tools. This comprised concepts of triage tool structure, intended population and setting, inclusion and exclusion criteria, and included variables and thresholds. Triage tools were coded by a single researcher and checked independently by a second investigator. Analogous concepts were grouped together under a single code (e.g. ‘inhalational injury’ and ‘respiratory tract burns’). Variables with differing thresholds/cut points or representing meaningfully different concepts were coded separately (e.g. ‘>20%’ versus ‘>30% body surface area burns’, ‘spinal injury with neurological deficit’ versus ‘spinal injury with paralysis’). Composite triage tool criteria, consisting of multiple separate concepts grouped together, were split into their constituent terms if they represented distinctly different conceptions (e.g. crushed, mangled or degloved extremity). Coding units were summarised using tabulation, descriptive statistics and visualisation using hierarchical scaled treemaps (Chazard et al., 2006).

Thematic analysis was also performed to identify additional higher-level patterns within the data, with emerging themes becoming categories for analysis (Kiger & Varpio, 2020). Coding framework and category construction was determined inductively based on the data’s characteristics by a single researcher and checked by a second investigator. A narrative synthesis of findings was then undertaken.

This study was undertaken as part of the Major Trauma Triage Study (MATTS) funded by the National Institute of Health Research Health Technology Agency Assessment Programme (NIHR HTA ref: 17/16/04). A study protocol was pre-specified. Ethical approval was provided by Yorkshire and The Humber ‒ Bradford Leeds Research Ethics Committee (Reference: 19/YH/0197).

## Results

All UK ambulance services and major trauma networks provided information on their major trauma triage tools between 2012 and 2021. A literature search revealed five systematic reviews examining major trauma triage tools during the same study period (see Supplementary 1) (Fuller et al., 2021; Gianola et al., 2021; van der Sluijs et al., 2018; van Rein et al., 2017; van Rein et al., 2018). In total, 53 major trauma tools were subsequently included for analysis, comprising 19 UK tools and 34 published international tools (detailed in Supplementary 1). Of these, 34 were adult-only tools, 11 were adult focused (primarily designed for adults with minor modifications for use in children), six were paediatric only and two were for elderly patients only. Age thresholds for non-adult tools ranged from <16 to <12 years for paediatric and >65 to >70 years for geriatric tools.

The structure and format of the identified tools is summarised in Table 1. Most triage tools were developed by expert opinion and were paper based with no evidence of any data-modelling process apparent (n = 42/53, 80%), although some were also available in electronic format (e.g. within electronic patient report forms or mobile application). A common structure of multiple domains (or ‘steps’) was evident for these tools, with constituent triage predictors assessed in parallel as a ‘checklist’. Physiology and anatomical injury steps were present in all such tools (n = 42/42, 100%), representing mandatory bypass/pre-alert criteria. Mechanism of injury (n = 31/42, 74%) and ‘special circumstance’ (patient or trauma system considerations, e.g. anticoagulant use, concomitant burn injury, n = 18/42, 43%) steps were less commonly implemented and, when present, were usually included as discretionary variables. A minority of tools were statistically derived prediction models, operationalised either as simple scores (n = 10/53, 19%) or as an electronic application (n = 1/53, 1%), providing a probability or total number for the likelihood of major trauma. All models included physiology variables, but anatomical (n = 6/11, 55%), mechanistic (n = 2/11, 18%) and special circumstance (n = 2/11, 18%) predictors were less commonly included. Triage tools otherwise differed widely, with variation in the intended population, individual variables and cut points and formal incorporation of clinical judgement. Most UK tools included an option to obtain remote senior clinical advice (n = 16/19, 84%), which varied from consultation with an MTC emergency medicine consultant (n = 1/16, 6%) to a dedicated ambulance service ‘trauma desk’ or ‘critical care’ desk staffed by specialist or non-specialist paramedics (n = 15/16, 94%). Senior clinical advice was less commonly incorporated into international tools (14/34, 41%), comprising remote physician advice available from the ambulance service ‘medical control’.

**Table 1.** Triage tool structure and format.

Triage tool structure		n/N	%
Derivation/format	Variable checklist^a^	42/54	80%
	Statistical prediction model (score)	10/54	19%
	Statistical prediction model (electronic application)	1/54	1%
Target age group	Adult only	35/54	66%
	Adult focused	11/54	21%
	Paediatric	5/54	9%
	Elderly	2/54	4%
Intended population defined	Serious injuries	3/54	6%
	Suspected major trauma	8/54	15%
	Significant mechanism	1/54	2%
	Serious injuries or significant mechanism	1/54	2%
	Suspected major trauma, except nursing home residents or CFS>4 excluded	1/54	2%
	Injury	36/54	66%
	Not defined		
Variable domains present	Physiology	54/54	100%
	Anatomical injury	48/53	91%
	Mechanism of injury	33/53	62%
	Special circumstances	20/53	38%
Unstructured clinical judgement	Formally incorporated	18/53	34%
Decision support	Option for senior clinical advice	15/53	28%

^a^Variable checklists were derived through expert opinion and available as paper/hard-copy versions, embedded within electronic patient report forms or mobile applications.

**Figure 1:**
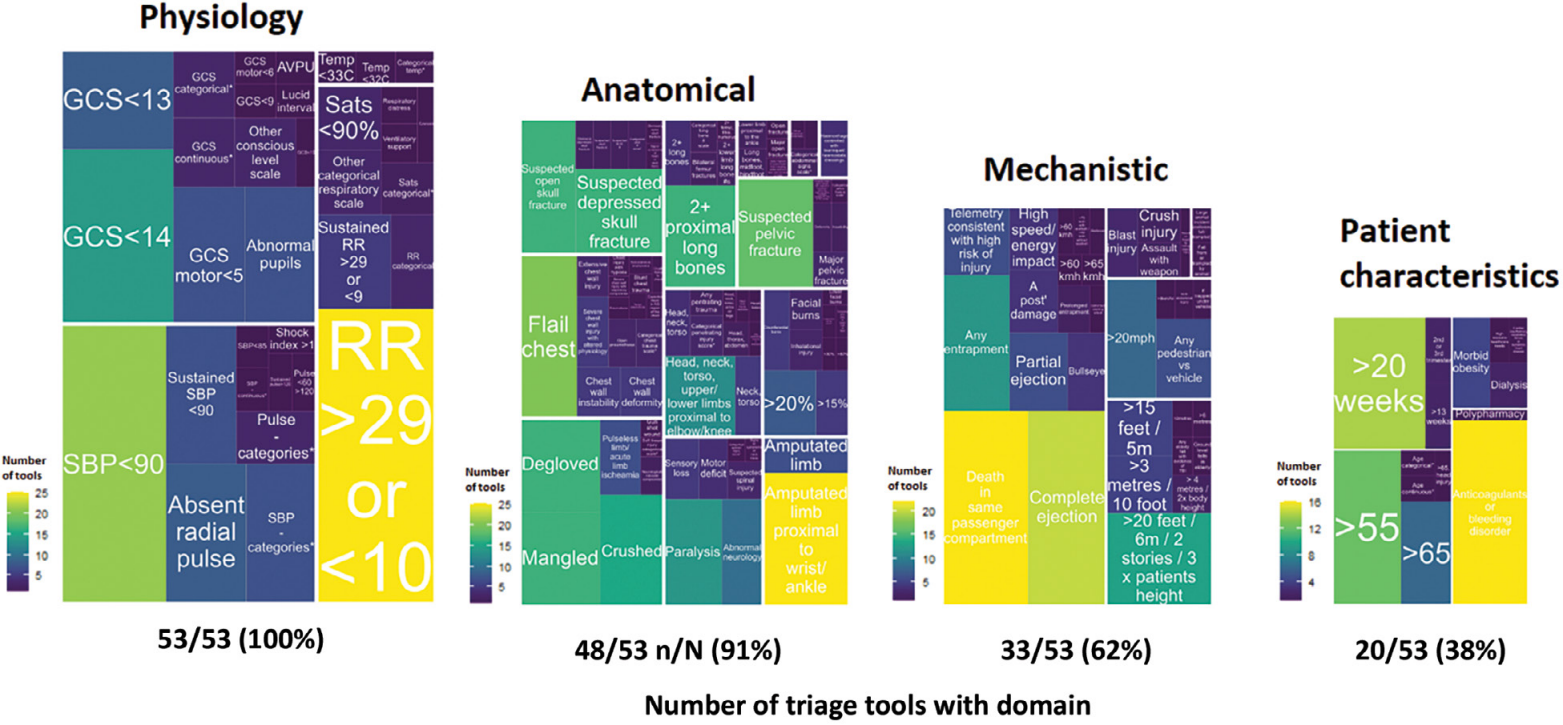
Figure 1. Scaled treemaps demonstrating how commonly domians and individual variables are used across UK and international triage tools.

Individual triage variables from identified adult triage tools (n = 45) are summarised in Figure 1. In total, 173 distinct variables were used, and the median number of constituent triage variables in each triage tool was 19 (range 3‒31). Variable descriptions ranged from precise to vague terms, within and across triage tools (e.g. ‘Suspected pelvic fracture’, compared with ‘Suspected major pelvic fracture, where mechanism of injury is suggestive of a pelvic fracture AND is accompanied by any one or more of the following: haemodynamic instability/signs of shock; deformity on examination; suspected open pelvic fracture due to bleeding PU, PV or PR (or scrotal haematoma)’). Cardiovascular, respiratory and level-of-consciousness physiological variables were included in all tools, with moderate variation evident in the exact definitions and cut points used. Anatomical injury variables were less common (n = 41/46 tools, 89%) and more diverse, with chest injury (89%), penetrating trauma (76%), spinal injury (72%), pelvic injury and limb amputation (72%) being the commonest categories; however, the exact number and definitions differed greatly across tools. When present, there was very wide variation in the number and definitions of mechanism and special circumstances variables. Paediatric and elderly triage tools demonstrated similar findings.

The size of each individual treemap is proportional to how often that domain of variables was included in triage tools. Within each treemap, the size and colour of each block indicates how commonly an individual variable was observed, e.g. a physiology domain was included in all triage tools, with respiratory rate <10 and >29, SBP <90 and GCS <14 being the commonest individual variables.

Three distinct patterns of triage tools were identified during thematic analysis: ‘specific and prescriptive’, ‘sensitive and permissive’ and ‘intermediate’. These three groups were similarly sized, but differed in terms of intended population, number of triage variables, thresholds, scope for clinical judgement and relative diagnostic accuracy. ‘Specific and prescriptive’ tools defined a higher bar for application, with inclusion criteria restricting use to high-energy mechanisms of injury or suspected major trauma. They included only physiological and anatomical steps, contained relatively fewer variables and dictated stricter, more conservative variable cut points or descriptors. Use of clinical judgement was uncommon, with less provision of remote clinical support. Together these characteristics would result in relatively higher specificity and a lower ratio of false positive to false negative cases. Rural trauma networks, with increased travel times, were over-represented within this group.

In contrast, ‘sensitive and permissive’ tools did not define inclusion criteria, potentially allowing application to a wider range of injured patients. The number of steps was increased, with mechanism of injury and special circumstance domains also present. More variables were included within each step, and more liberal cut points and definitions were used. Unstructured clinical judgement and remote clinical advice, over and above the included variables, were commonly integrated. Together these differences would result in relatively higher sensitivity, favouring false positive over false negatives cases. Triage tools from urban and more densely populated areas were more common within this grouping.

**Figure 2:**
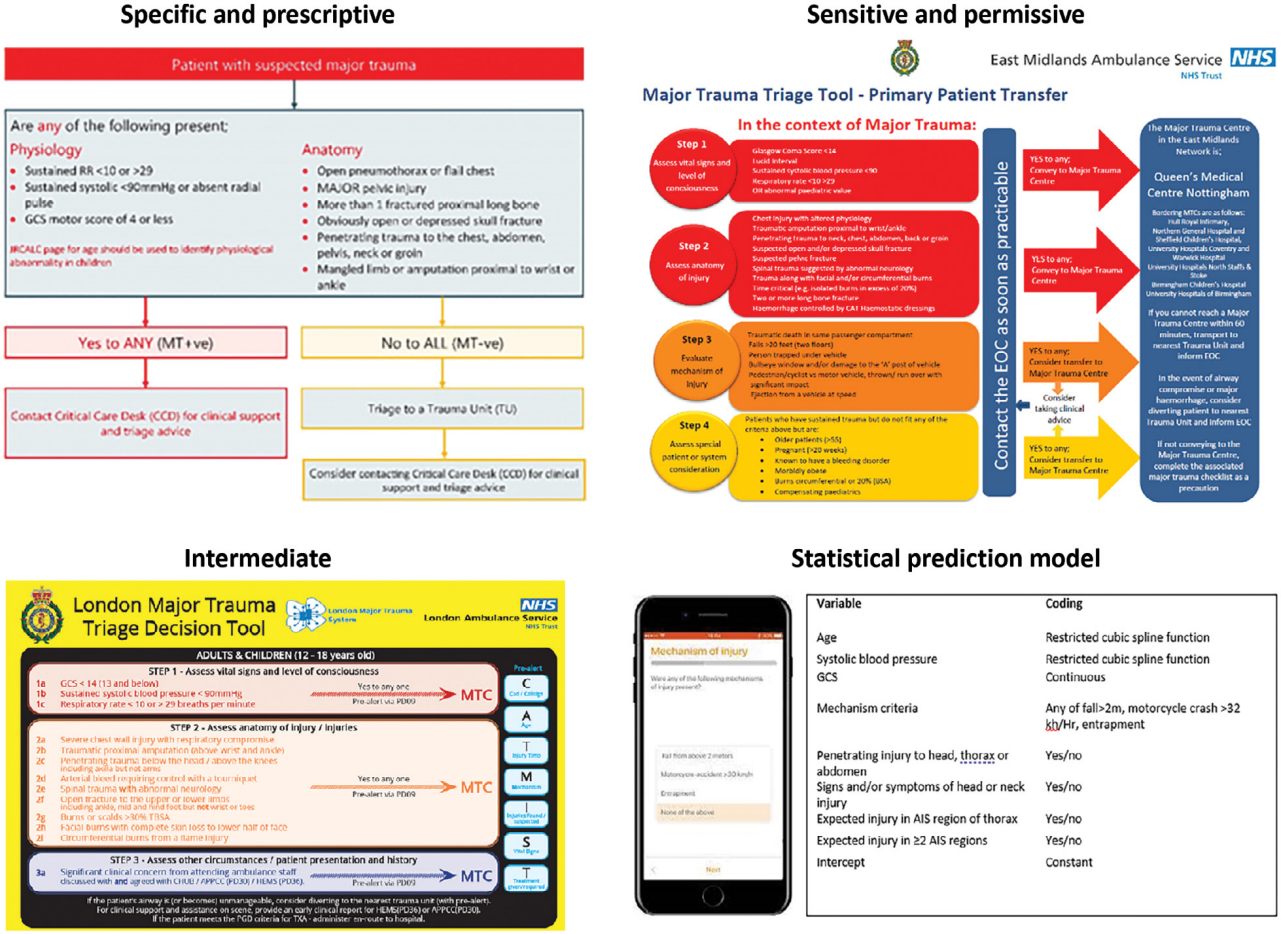
Figure 2. Exemplar triage tools for each thematic category.

An intermediate group of triage tools displayed characteristics in between the sensitive and specific triage tools. These tools did not delineate an intended population, comprised only physiology and anatomical steps, explicitly included a role for clinical judgement and included variable numbers and cut points interposed between the two preceding groups. The smaller number of prediction models presented a distinct grouping, using weighted variables added together to provide a score or probability for major trauma. The trade-off between sensitivity and specificity could subsequently be manipulated by setting a score or probability threshold for positivity. These models included relatively fewer variables, similar to those present in the specific triage tools. Clinical judgement and senior clinical support were not integrated. These different groups are summarised in Table 2. Exemplar triage tools for each thematic group are presented in Figure 2.

**Table 2.** Triage tool subgroups.

Property	Specific and prescriptive	Sensitive and permissive	Intermediate	Statistical prediction models
UK tools	5	7	4	0
Derivation	Expert opinion	Expert opinion	Expert opinion	Statistical modelling
Format	Checklist of variables^a^	Checklist of variables^a^	Checklist of variables^a^	Clinical score or prediction equation^b^
Inclusion	High-energy mechanism of injury or suspected major trauma	Not defined	Inconsistently defined as suspected major trauma	Inconsistently defined as suspected major trauma
Domains (or ‘steps’)	2 Mandatory physiology and anatomical	4 Mandatory physiology and anatomical Discretionary mechanism and patient characteristics	3 Mandatory physiology and anatomical Unstructured clinical judgement	N/A Variables given weights and added together to provide a score or probability
Relative number of variables	Fewer	Higher	Intermediate	Fewer
Variable descriptions/ thresholds	Stricter	Liberal	Intermediate	Categorical or continuous scoring of variable
Relative accuracy	Specific	Sensitive	Intermediate	Intermediate
Formal clinical judgement	Less often	More often	More often	No
Senior clinical support	Less often	More often	More often	No
Other	Rural trauma networks	Includes all elderly and paediatric tools	—	—

^a^Presented as paper algorithms, embedded within the electronic patient record or, rarely, in electronic applications. ^b^Operationalised as paper scoring system or electronic scoring applications.

Triage tools used in inclusive trauma networks were distinctive in containing additional bypass exclusion criteria (e.g. travelling time limits and diversion to closer hospitals for patients with uncontrollable airway, breathing or circulation deficits). This group of tools were often formatted as flow charts, more commonly included the option for senior clinical support and frequently included triage variables for open fractures and burns. More recent tools, post 2020, tended to have fewer mechanism of injury or special circumstance criteria. No other major themes were identified in the data, and UK tools appeared to be broadly comparable with international tools, except for increased provision of senior clinical decision support.

## Discussion

In summary, a wide range of UK and international major trauma triage tools were identified, with marked variation in structure and included variables. Four broad groups were identified, comprising expert-derived specific and prescriptive, sensitive and non-prescriptive, intermediate tools and statistically derived prediction models. Triage tools used in inclusive trauma networks were notable for additional bypass exclusion criteria and increased use of senior clinical decision support. More recently introduced tools tended to de-emphasise mechanism of injury and patient characteristic variables.

The US Field Triage Decision Scheme (FTDS) was initially introduced by the American College of Surgeons Committee of Trauma in 2006, with further iterations published in 2011 and 2021 (Newgard et al., 2022; Sasser et al., 2009, 2012). It is the most studied triage tool, and despite reported under- and over-triage of 14‒34% and 12‒31% respectively (van Rein et al., 2017), it might be considered the ‘industry standard’. Its structure of multiple domains (or ‘steps’), comprising physiology, injury, mechanistic and patient characteristic variables, has been influential and is followed by the majority of identified UK and international tools (Mackersie, 2006). The FTDS is a ‘sensitive and non-prescriptive’ tool, which appeared to have been modified to provide additional instructions and to increase specificity when introduced to other settings.

The central content of major trauma triage tools is the constituent variables and the relationship between them. A very wide range of variables and cut points were identified in the current study. No triage tool provided variable definitions, and there were often subtle differences between variable labels (e.g. ‘thorax and abdomen’ versus ‘trunk’, or ‘obviously’ versus ‘suspected’ depressed skull fracture). Not all anatomical domains were included, with blunt abdominal injuries, for example, rarely included. No point-of-care tests were used, and future investigation is required to investigate whether ultrasound (El Zahran & El Sayed, 2018), lactate (Robinson, 2019), end tidal CO_2_ (Wilson et al., 2021) or other novel predictors have utility. The observation that newer triage tools tended to exclude mechanism of injury or special circumstance variables may reflect an emerging perception that these factors have less accuracy for major trauma (Boyle, 2007; Moriarty et al., 2021; Stuke, Duchesne, Greiffenstein, et al., 2013; Stuke, Duchesne, Hunt, et al., 2013).

Clinical judgement may be used at various levels within pre-hospital major trauma triage, including the decision to use a triage tool, interpretation of variable definitions, use of discretionary variables or planned application of unstructured clinical judgement. Clinical acumen has been repeatedly demonstrated to outperform strict adherence to clinical decision rules across many medical conditions (Sanders et al., 2015; Schriger et al., 2017). It is therefore not surprising that clinical judgement was formally incorporated into many triage tools, particularly given the heterogeneity of major trauma and often confounding pre-hospital circumstances. Decision support, provided by senior clinicians, for example from a dedicated ambulance service ‘trauma desk’, was used in many UK tools and could further finesse the benefit of clinical judgement.

It is well established that major trauma encompasses different disease entities in children, adults and the elderly (Coats & Lecky, 2017), with contrasting incidence, mechanisms, patterns of injury and treatment considerations. The finding of predominantly adult triage tools, with uncommon minor modifications for children and the elderly, might reflect the need to avoid complexity from multiple different tools or age-variable interactions in the challenging pre-hospital environment. However, dedicated variables and thresholds could have the potential to improve accuracy, subject to robust study.

Most identified tools were created through expert consensus; however, advances in health-data science, with access to large, robust linked datasets, may facilitate increased use of statistical prediction modelling and machine learning (Hastie et al., 2009; Steyerberg, 2009). This study provides a comprehensive source of candidate variables for use in these approaches. However, it is unclear whether less intuitive prediction models and black box machine-learning algorithms would be acceptable for deployment in complex trauma networks or demonstrate improved performance in the low signal to noise pre-hospital environment.

Strikingly, existing triage tools did not state their target definition of major trauma. Traditionally, an injury severity score (ISS) threshold of ≥16 has been used to define major trauma (Baker et al., 1974). However, whereas the ISS provides an overall estimate of injury severity, it does not account for heterogeneity in the intensity, urgency and complexity of treatments required for different injuries. Certain patients with high ISS, but not requiring urgent resuscitation or specialist interventions, may be satisfactorily managed entirely in a non-MTC or with a later planned secondary transfer if necessary. Resource-based criteria may be a better target in triage tool design.

Where defined, the intended patient population for application was high-energy trauma or suspicion of major trauma. However, ‘stealth trauma’ is an increasingly recognised phenomenon, with a significant proportion of severe injuries occurring with low-energy mechanisms or presenting atypically (Davies & Coats, 2020). Notably, UK triage tools used in inclusive trauma networks generally contained additional instructions on triage tool use, including bypass exclusions, over and above a checklist of triage variables.

Identified triage tools also did not explicitly define their desired sensitivity/specificity trade-off. Influenced by the FTDS, earlier tools prioritised sensitivity over specificity, with higher numbers of variables and more liberal variable thresholds. Conversely, more recently developed tools appeared to favour specificity. This could reflect emerging evidence from economic evaluations that high sensitivity triage is not cost-effective due to the large number of false positive cases unnecessarily pre-alerted and transported to MTCs (Newgard et al., 2013, 2016; Pollard et al., 2022). Additionally, several UK ambulance services (e.g. London Ambulance Service) introduced revised triage tools in response to the COVID-19 epidemic, to prevent MTC emergency departments becoming overwhelmed. Interestingly, these triage tools have continued in use following abatement of the pandemic, suggesting satisfactory performance.

A single scoping review examining pre-hospital triage tools has been published previously but did not focus on major trauma (Bhaumik et al., 2022). No other previous studies were found that have qualitatively investigated major trauma triage tool structure and content. This study has several strengths, including a comprehensive search strategy, concordance with document analysis recommendations and detailed coding of triage tools. However, limitations could include a non-exhaustive search for international triage tools, only searching the Medline database and checking of triage tool coding, rather than independent re-coding. The inclusion of all recent NHS tools ensures the findings are directly applicable to UK major trauma triage. Included international tools were exclusively from the developed world (Australia, Europe, Hong Kong or the US), and external validity to middle- and lower-income settings is therefore uncertain.

## Conclusion

In conclusion, many diverse major trauma triage tools were identified, with no consensus in format, structure or content. Quantification of constituent variables will provide a comprehensive source of candidate predictors for consideration in future triage tools, prediction models or machine-learning algorithms. Other important considerations include providing variable definitions, the addition of supporting operational information and defining the entry threshold for tool use, optimal sensitivity/specificity trade-off and target condition.

## Acknowledgements

We would like to acknowledge members of the MATTS study management group team for their general support and advice, including James Baird, Richard Pilbery, Natalie Kean, Fiona Lecky, Antoinette Edwards, Andy Rosser, Rachael Fothergill, Sarah Black, Fiona Bell, Michael Smyth, Jason Smith, Gavin Perkins, Esther Herbert, Stephen Walters, Cindy Cooper, Ian Maconochie, Mathew Ward, Mark Millins, Emily Turton, Simon Waterhouse, Matt Stevenson, Daniel Pollard, Abdullah Pandor, Maria Robinson, Stuart Reid and Di Charles.

## Author contributions

GF conceived the study. GF and SK managed the study. GF and CH performed the searches, data collection and coding. All authors made substantial contributions to study design, data interpretation and article writing. All authors had full access to all the data in the study and can take responsibility for the integrity of the data and the accuracy of the data analysis. JT acts as the guarantor for this article.

## Conflict of interest

None declared.

## Ethics

Not required.

## Funding

None.
